# Alcohol and drug comorbidity among survivors of physical injuries receiving mandated screening and brief intervention at a level-I trauma center

**DOI:** 10.1186/1940-0640-7-S1-A3

**Published:** 2012-10-09

**Authors:** Douglas Zatzick, Dennis Donovan, Gregory Jurkovich, Frederick Rivara, Chris Dunn, Rick Ries, Larry Gentillelo

**Affiliations:** 1 Harborview Injury Prevention and Research Center, University of Washington School of Medicine, Seattle, WA, USA; 2 Southwestern Medical Center, University of Texas, Dallas, TX, USA

## 

The American College of Surgeons Committee on Trauma (ACS/COT) has mandated alcohol screening and brief intervention (SBI) for all level-I trauma centers. Few investigations have assessed alcohol and drug comorbidity among patients receiving mandated alcohol SBI at trauma centers. In this study, 878 randomly selected level-I trauma center inpatients were systematically screened for alcohol and drug use problems with blood and urine toxicology laboratory results and self-report questionnaire items. Patients were systematically screened for alcohol use by blood alcohol concentration (BAC) testing and administration of the Alcohol Use Disorders Identification Test-Consumption (AUDIT-C). Screening for stimulant use (i.e., amphetamines and cocaine) included urine toxicology testing and single-item self-report. Screening for marijuana use included urine testing and single-item self-report. Screening for prescription and nonprescription opioid use included single-item self-report only. Fifty percent of patients (435/878) screened positive for problem alcohol use. Approximately 20% screened positive for cocaine use, 7.7% for amphetamine use, 7.5% for opioid use, and 37% for marijuana use. Among the 50% of patients who screened positive for problem alcohol use, 61.1% had one or more drug comorbidities. Of all 878 patients in the sample, 166 were seen by the trauma center’s addiction intervention service for mandated alcohol SBI. Of these, 33% were positive for problem alcohol use only, 44% were positive for alcohol and other drug use, 12% were positive for marijuana, stimulants, or opioid use only, and 11% screened negative for both alcohol and drugs. The majority of patients receiving mandated alcohol SBI at a level-I trauma center screened positive for comorbid use of one or more drugs. Clinical SBI research protocols that realistically account for alcohol and drug comorbidity are needed to inform the development of ACS/COT SBI guidelines.

